# Glucose metabolism and autonomic function in healthy individuals and patients with type 2 diabetes mellitus at rest and during exercise

**DOI:** 10.1113/EP091444

**Published:** 2023-12-05

**Authors:** Takuto Hamaoka, Urs A. Leuenberger, Rachel C. Drew, Matthew Murray, Cheryl Blaha, Jonathan Carter Luck, Lawrence I. Sinoway, Jian Cui

**Affiliations:** ^1^ Penn State Heart and Vascular Institute Pennsylvania State University College of Medicine Hershey Pennsylvania USA; ^2^ Department of Exercise and Health Sciences University of Massachusetts Boston Boston Massachusetts USA

**Keywords:** heart rate variability, muscle sympathetic nerve activity, type 2 diabetes mellitus

## Abstract

Autonomic dysfunction is a common complication of type 2 diabetes mellitus (T2DM). However, the character of dysfunction varies in different reports. Differences in measurement methodology and complications might have influenced the inconsistent results. We sought to evaluate comprehensively the relationship between abnormal glucose metabolism and autonomic function at rest and the response to exercise in healthy individuals and T2DM patients. We hypothesized that both sympathetic and parasympathetic indices would decrease with the progression of abnormal glucose metabolism in individuals with few complications related to high sympathetic tone. Twenty healthy individuals and 11 T2DM patients without clinically evident cardiovascular disease other than controlled hypertension were examined. Resting muscle sympathetic nerve activity (MSNA), heart rate variability, spontaneous cardiovagal baroreflex sensitivity (CBRS), sympathetic baroreflex sensitivity and the MSNA response to handgrip exercise were measured. Resting MSNA was lower in patients with T2DM than in healthy control subjects (*P* = 0.011). Resting MSNA was negatively correlated with haemoglobin A_1c_ in all subjects (*R* = −0.45, *P* = 0.024). The parasympathetic components of heart rate variability and CBRS were negatively correlated with glycaemic/insulin indices in all subjects and even in the control group only (all, *P* < 0.05). In all subjects, the MSNA response to exercise was positively correlated with fasting blood glucose (*R* = 0.69, *P* < 0.001). Resting sympathetic activity and parasympathetic modulation of heart rate were decreased in relationship to abnormal glucose metabolism. Meanwhile, the sympathetic responses to handgrip were preserved in diabetics. The responses were correlated with glucose/insulin parameters throughout diabetic and control subjects. These results suggest the importance of a comprehensive assessment of autonomic function in T2DM.

## Abstract (highlights)

- **What is the central question of this study?**
  Are sympathetic and parasympathetic nerves at rest and during exercise equally impaired in type 2 diabetes mellitus? What is the relationship of these dysfunctions to abnormal glucose metabolism?
- **What is the main finding and its importance?**
  Resting sympathetic activity and parasympathetic modulation of heart rate were decreased in relationship to abnormal glucose metabolism. Meanwhile, the sympathetic responses to handgrip were preserved in diabetics. The responses were correlated with glucose/insulin parameters in diabetics and control subjects. These results suggest the importance of a comprehensive assessment of autonomic function in diabetics.

## 1 INTRODUCTION

Autonomic dysfunction is a common complication in patients with diabetes mellitus (DM). Both sympathetic (Grassi et al., 2020; Huggett et al., 2003; Straznicky et al., 2012) and parasympathetic autonomic dysfunction (Benichou et al., 2018) have been reported in DM and have been associated with cardiovascular complications and an adverse prognosis (Pop‐Busui et al., 2010; Smith et al., 2004). In previous reports, parasympathetic nerve activity measured by the high‐frequency (HF) component of heart rate variability (HRV) decreased with disease progression and was related to the severity of the abnormal glucose/insulin metabolism in type 2 diabetes mellitus (T2DM) (Spallone, 2019). Sympathetic activity has been assessed previously in T2DM via measures of muscle sympathetic nerve activity (MSNA) (Heusser et al., 2022; Holwerda et al., 2016a, 2016b; Huggett et al., 2005, 2003; Straznicky et al., 2012), the concentration of circulating noradrenaline (Caviezel et al., 1982; Straznicky et al., 2012) and the ratio of the low‐frequency (LF) component to the HF component of HRV (i.e., LF/HF) (Benichou et al., 2018; Lee et al., 2020). Yet, few studies evaluated HRV and MSNA simultaneously. Resting MSNA in T2DM patients was reported to be higher (Huggett et al., 2005, 2003; Straznicky et al., 2012) in many previous reports. Meanwhile, some studies showed that MSNA in T2DM was not different from that of control subjects (Heusser et al., 2022; Holwerda et al., 2016a, 2016b). Moreover, LF/HF and plasma noradrenalin were decreased in comparison to non‐diabetic subjects (Benichou et al., 2018; Caviezel et al., 1982). Therefore, the character of sympathetic nerve dysfunction in T2DM remains controversial. Furthermore, differences in the progression of the sympathetic and parasympathetic autonomic dysfunction and the relationship with glycaemic/insulin indices have not been elucidated fully.

Abnormal baroreflex control of heart rate (HR) (cardiovagal baroreflex sensitivity, CBRS) (Kück et al., 2020; Sakamoto et al., 2019) has been reported in T2DM and is associated with adverse cardiovascular events (Sakamoto et al., 2019). However, other studies showed preserved CBRS (Huggett et al., 2005) or altered CBRS when related to obesity (Holwerda et al., 2016b) but not to T2DM itself. Baroreflex control of MSNA (sympathetic baroreflex sensitivity, SBRS) in T2DM was not decreased in that study (Holwerda et al., 2016b). Thus, the effects of T2DM on baroreflex function are still controversial. Importantly, to our knowledge, it has been unclear whether the changes in baroreflex function in T2DM are related to glycaemic/insulin indices.

Therefore, the purpose of this study was to evaluate sympathetic (i.e., MSNA) and parasympathetic (i.e., HRV) nerve activity and BRS (both SBRS and CBRS) and to evaluate their relationship with glycaemic/insulin indices in patients with T2DM and in control subjects. We also assessed the blood pressure (BP) and MSNA responses to handgrip (HG) exercise. Cardiovascular disease itself (e.g., congestive heart failure; Seravalle et al., 2019), complicated by T2DM, can increase MSNA, making it difficult to demonstrate the direct effects of abnormal glucose/insulin metabolism on autonomic function. Muscle sympathetic nerves consist of small and unmyelinated postganglionic fibres (Vallbo et al., 2004). It seems possible that muscle sympathetic nerves are injured by T2DM before the manifestation of symptomatic neuropathy but that this is concealed by sympatho‐excitatory cardiovascular complications. Therefore, in this study, we included T2DM patients without obvious cardiovascular complications. We hypothesized that in addition to the parasympathetic nervous dysfunction, sympathetic activity at rest and the response to exercise would be decreased in T2DM.

## 2 MATERIALS AND METHODS

### 2.1 Subjects

Eleven patients with T2DM (reported duration of the disease: 2.6 ± 2.3 years) were included in this study (Table 1). Twenty healthy individuals matched for age and sex were included as control subjects (Table 1). Individuals with diseases requiring regular hospital visits or medications were excluded from the control group. Mild hyperglycaemia, which does not meet diagnostic criteria for diabetes (i.e., pre‐DM), and obesity were not excluded from the control group. Healthy individuals whose resting MSNA could not be recorded were excluded at that point. Patients with T2DM had received medications (Table 2). Subjects completed a health history questionnaire and underwent a fasting blood test to measure fasting blood glucose (FBG), insulin, haemoglobin A_1c_ (HbA_1c_) and homeostatic model assessment of insulin resistance (HOMA‐IR). Haemoglobin A_1c_ (i.e., glycated haemoglobin) reflects the average blood glucose level for the past 2–3 months and represents a standard parameter to diagnose DM and monitor glucose control. Those T2DM patients with clinically evident cardiovascular diseases, including coronary/cerebral artery disease, heart failure, pulmonary hypertension, renal failure and untreated sleep‐disordered breathing, were excluded. Patients with symptomatic neuropathy were also excluded. Those T2DM patients with essential hypertension who had already received treatment were not excluded (*n* = 5) (Table 2). Owing to the limited number of patients who met the inclusion criteria, in the event that the resting MSNA could not be recorded in a T2DM patient, the study was still continued. To obtain an adequate range of HbA_1c_ in healthy subjects, their number was double that of the T2DM patients in this study.

**TABLE 1:** Group characteristics.

Characteristic	Control (*n* = 20)	T2DM (n = 11)	PScontrol (*n* = 9)
Age, years	58.3 ± 5.6	54.0 ± 8.9	58.6 ± 6.9
Male, *n* (%)	12 (60)	7 (64)	7 (78)
BMI, kg/m^2^	26.6 ± 5.4^*^	31.5 ± 6.4	29.8 ± 6.0
SBP, mmHg	116.5 ± 6.8^*^	126.2 ± 13.9	115.1 ± 8.1
DBP, mmHg	76.6 ± 5.1	78.6 ± 8.0	76.7 ± 4.7
HR, beats/min	60.5 ± 5.2	65.0 ± 9.6	60.7 ± 5.3
Respiratory rate, breaths/min	16.1 ± 3.0	16.6 ± 2.7	17.6 ± 3.0
HbA_1c_, %	5.6 ± 0.3^*^	6.8 ± 0.9	5.4 ± 0.2^*^
HOMA‐IR	1.5 ± 0.7^*^	5.0 ± 3.8	1.7 ± 0.8^*^
Insulin, mIU/L	6.6 ± 2.6^*^	15.1 ± 10.3	7.4 ± 3.2^*^
FBG, mg/dL	90.1 ± 9.1^*^	132 ± 24.2	91.2 ± 9.2^*^
SDNN, ms	52.2 ± 18.5^*^	35.8 ± 9.0	63.2 ± 20.0^*^
RMSSD, ms	32.1 ± 12.0	31.1 ± 17.8	35.0 ± 16.0
LF, ms^2^	669.1 ± 400.0^*^	335.6 ± 171.1	785.0 ± 505.7^*^
HF, ms^2^	414.0 ± 456.3	321.1 ± 287.1	533.5 ± 628.0
LF/HF	2.8 ± 2.4	1.9 ± 1.3	3.3 ± 3.1
MSNA‐BF, bursts/min	34.0 ± 10.0^*^	23.8 ± 8.5	33.1 ± 8.4^*^
MSNA‐BI, bursts/100 heart beats	56.7 ± 19.0^*^	37.1 ± 17.1	57.2 ± 20.2^*^
CBRS, ms/mmHg	14.5 ± 6.3^*^	8.9 ± 4.3	14.9 ± 6.4^*^
SBRS‐BI, bursts/100 heart beats/mmHg	−2.8 ± 1.6	−2.3 ± 1.4	−2.4 ± 1.0
SBRS‐Total MSNA, units/beat/mmHg	−0.7 ± 0.4	−0.6 ± 0.4	−0.6 ± 0.2

*Note*: Values are the mean ± SD. The FBG, insulin and HOMA‐IR of one T2DM patient were excluded because it was not a fasting blood sample (HbA_1c_ was included). The MSNA was recorded successfully in eight patients with T2DM and 20 control subjects. The HRV was calculated successfully in 10 patients with T2DM and 18 control subjects. Resting CBRS was assessed successfully in 10 T2DM patients and in 16 control subjects. Resting SBRS was assessed successfully in eight T2DM patients and in 16 control subjects.

Abbreviations: BF, burst frequency; BI, burst incidence; BMI, body mass index; CBRS, cardiovagal‐baroreflex sensitivity; DBP, diastolic blood pressure; FBG, fasting blood glucose; HF, high frequency; HOMA‐IR, homeostatic model assessment of insulin resistance; HR, heart rate; LF, low frequency; MSNA, muscle sympathetic nerve activity; PScontrol, propensity score matched control subjects; RMSSD, root mean square of successive R–R interval differences; SBP, systolic blood pressure; SBRS, sympathetic baroreflex sensitivity; SDNN, standard deviation of normal‐to‐normal intervals; T2DM, type 2 diabetes mellitus.

* *P* < 0.05 compared with the T2DM group.

**TABLE 2:** Complications and medications in patients with type 2 diabetes mellitus.

Parameter	T2DM (*n* = 11)
Hypertension, *n* (%)	5 (45.5)
Dyslipidaemia, *n* (%)	6 (54.5)
Sulfonylurea, *n* (%)	1 (9.1)
Biguanide, *n* (%)	6 (54.5)
Insulin, *n* (%)	1 (9.1)
α‐Glucosidase inhibitor, *n* (%)	0 (0)
GLP‐1 receptor agonist, *n* (%)	0 (0)
SGLT2 inhibitor, *n* (%)	0 (0)
Statins, *n* (%)	6 (54.5)
ARB/ACEI, *n* (%)	5 (45.5)
Calcium channel blocker, *n* (%)	1 (9.1)
Diuretics, *n* (%)	0 (0)
β‐Blocker, *n* (%)	0 (0)
α‐Blocker, *n* (%)	0 (0)

Abbreviations: ACEI, angiotensin‐converting enzyme inhibitor; ARB, angiotensin receptor blocker; GLP‐1, glucagon‐like peptide‐1; SGLT2, sodium–glucose cotransporter 2; T2DM, type 2 diabetes mellitus.

All protocols were approved by the Institutional Review Board of the Penn State Milton S. Hershey Medical Center (Study00000120) and conformed with the *Declaration of Helsinki*, except for registration in a database. All participants provided written informed consent. Each subject received an explanation of the purpose, the protocol and the risks associated with the study before providing consent.

### 2.2 Measurements

As described in our previous report (Cui et al., 2006), beat‐by‐beat HR and BP (Finometer) at rest and during isometric HG exercise were recorded. After HG, postexercise muscle ischaemia (PEMI) was evaluated to examine the effect of metabolites produced by the exercise on the pressor reflex (i.e., metaboreflex) independent of the impact of mechanical stimulation and central command by the exercise (Grotle et al., 2020; Ishizawa et al., 2021). Resting values of beat‐by‐beat BP were verified by the cuff pressure from the brachial artery. Respiration rate was monitored by piezoelectric pneumography. Postganglionic MSNA was recorded from a peroneal nerve using a tungsten electrode, as described previously (Cui et al., 2006). This allowed us to obtain pulse‐synchronous multi‐unit MSNA bursts that meet established criteria (Vallbo et al., 1979).

### 2.3 Experimental protocol

The subjects were asked to fast for 8 h before the visit and to refrain from caffeine, alcohol and strenuous exercise for 24 h before the visit. Subjects who were following a medical regimen were asked to postpone taking the medications on the study day until completion of the study. Next, the maximal voluntary contraction of the non‐dominant hand was measured in the supine position. Microneurography was performed to obtain the MSNA recording (∼30 min). After an acclimation period, 5 min of baseline data were recorded. Thereafter, subjects performed isometric HG exercise at 30% maximal voluntary contraction until fatigue, followed by 2.5 min of PEMI by inflating a cuff on the upper arm to 250 mmHg. The final minute of recording during HG exercise was used to represent the HG response.

### 2.4 Data analysis

Data were collected with a data acquisition system (MacLab; ADInstruments, Castle Hill, NSW, Australia). The MSNA was evaluated as previously described (Cui et al., 2006). The number of MSNA bursts per minute [burst frequency (BF), in bursts per minute] and per 100 heart beats [burst incidence (BI), in bursts per 100 heart beats] were calculated. The amplitude of the integrated MSNA traces was normalized according to the baseline recording (Cui et al., 2006). Then the burst area of the integrated neurogram in a cardiac cycle was calculated, and the sum of the area per minute was used as Total MSNA (in units per minute).

Short‐term HRV (5 min) was analysed from the beat‐by‐beat R–R intervals using LabChart software (v.8; ADInstruments). The standard deviation of normal R–R intervals (SDNN), root mean square of successive R–R interval differences (RMSSD), absolute (in milliseconds square) and relative power (in normalized units) of LF (0.04–0.15 Hz), HF (0.15–0.45 Hz) and LF/HF were measured as HRV indices (1996). Premature ventricular/atrial contractions and electrical artefacts were excluded from the analysis. If stable high‐quality continuous recording segments of sinus rhythm were <5 min, the data were excluded from the HRV analyses.

The slope of the relationship between systolic BP (SBP) and cardiac R–R interval was calculated as CBRS using the sequence technique described previously (Hamaoka et al., 2021). The mean slope of all accepted sequences (i.e., *R* ^2^ > 0.8) was regarded as spontaneous CBRS (in milliseconds per millimetre of mercury) (Hamaoka et al., 2021). Spontaneous SBRS was calculated from the slope of the linear regression between diastolic BP (DBP) and MSNA. As described in previous reports (Hamaoka et al., 2021), the DBP for each cardiac cycle was grouped into bins with equal width (3 mmHg). Using all binned data, linear regression analyses between DBP and burst incidence or total MSNA were performed to evaluate SBRS (SBRS‐BI, in bursts per 100 heart beats per millimetre of mercury; SBRS‐Total MSNA, in units per beat per millimetre of mercury). For *R* values of the regression line ≥ 0.5, the slope was regarded as an acceptable SBRS slope (Hissen et al., 2018). As with HRV analysis, if stable high‐quality continuous recording segments (both beat‐by‐beat BP and ECG) were <5 min, the data were excluded from the BRS analyses.

### 2.5 Statistical analysis

Values are expressed as the mean ± SD. A priori power analysis was performed using G*Power v.3.1 (Faul et al., 2007). Based on a previous study (Kobayashi et al., 2010), to detect statistical significance in MSNA (BF) between two independent groups (T2DM vs. control) with a desired power of 0.8 and an α error of 5%, eight subjects in each group are required. All statistical analyses were performed using SPSS software (SPSS Science v.27.0; IBM). Student's unpaired *t*‐test was performed to compare differences between groups. Welch's *t*‐test was applied if the variance was heteroscedastic. The χ^2^ test was used to compare differences in the population rate between groups. Univariate regression analyses were performed to evaluate the linear relationship between glycaemic/insulin indices and autonomic or haemodynamic variables, including the response to HG exercise and PEMI (HRV, MSNA, HR and BP, and responses in these variables to HG exercise and PEMI). Two‐way mixed ANOVA (MANOVA), with time (baseline, HG and PEMI) as a within‐subject factor and the groups (T2DM and control) as a between‐subject factor, was performed to compare the changes in mean arterial pressure (MAP), HR and MSNA (BF, BI) by HG exercise and PEMI between groups. Because of the normalization procedure in calculating total MSNA, comparisons of total MSNA between groups are not recommended (Hart et al., 2017). Thus, total MSNA was excluded from two‐way MANOVAs. One‐way repeated‐measures ANOVA (RMANOVA) was performed to examine the changes in MAP, HR and MSNA (BF, BI and total MSNA) by HG exercise and PEMI within each group. To adjust for group differences in body mass index (BMI) and compare other variables, propensity score matching by BMI was performed. A calliper size (tolerance) of 0.045, a quarter of a standard deviation of the propensity score, was applied to the matching (Rosenbaum & Rubin, 1985). A value of *P* < 0.05 (two‐sided) was considered statistically significant.

## 3 RESULTS

Age and the proportion of males/females were not significantly different between groups. The BMI in the T2DM group was higher than in the control group (Table 1). One T2DM patient used insulin. Thus, the insulin and HOMA‐IR data for this patient were excluded from the mean value calculation and the regression analyses with other indices. The FBG and insulin data of one T2DM patient were excluded because they were not from a fasting blood sample, while the HbA_1c_ from this patient was included in the analysis. As expected, glycaemic and insulin indices in T2DM were higher than in control subjects (Table 1).

### 3.1 Resting haemodynamic variables and MSNA

**FIGURE 1:**
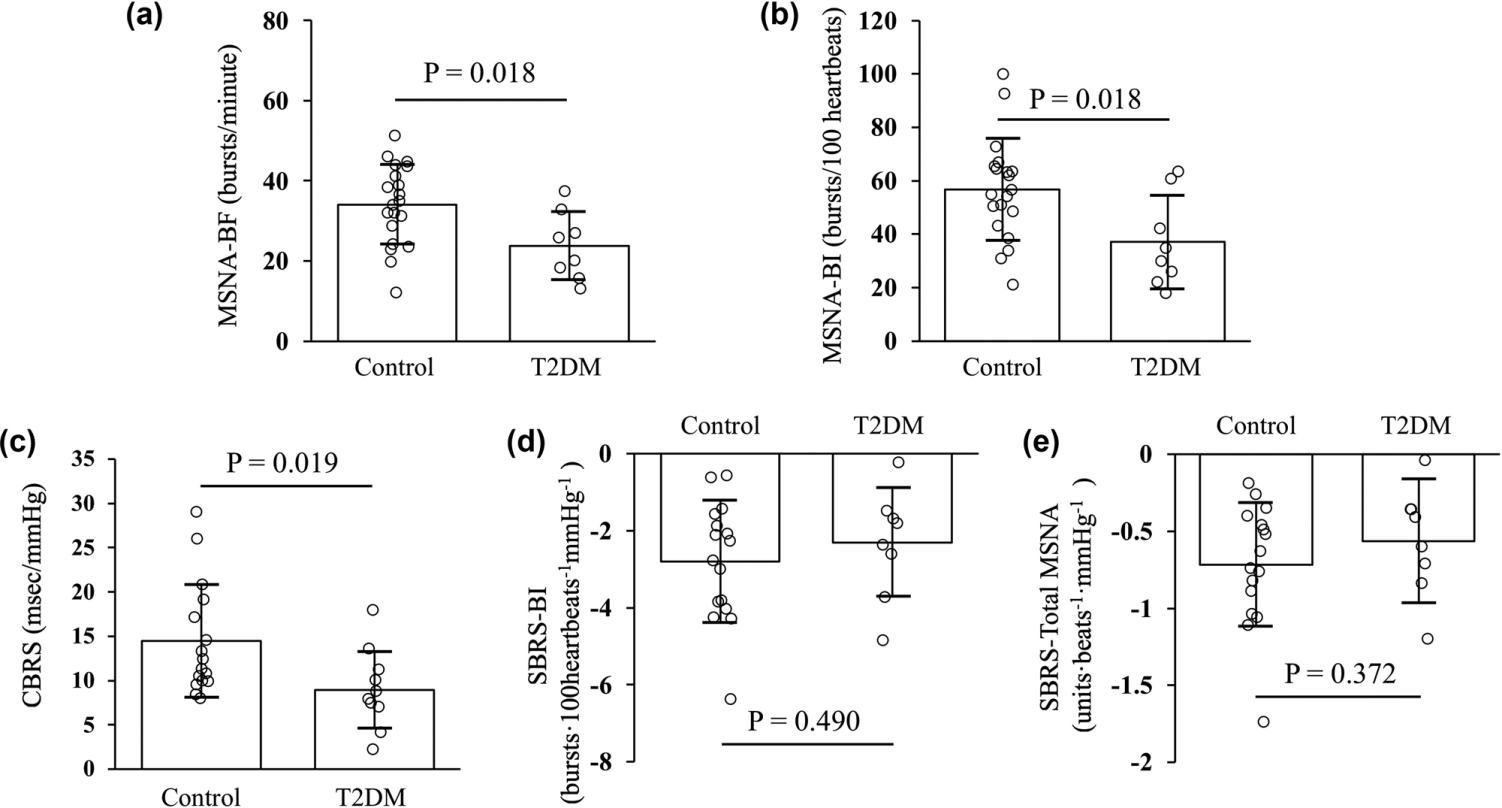
Comparisons of resting MSNA (a, BF; b, BI), CBRS (c), SBRS‐BI (d) and SBRS‐total MSNA (e) between groups. Small circles represent individual data. Abbreviations: BF, burst frequency; BI, burst incidence; CBRS, cardiovagal‐baroreflex sensitivity; MSNA, muscle sympathetic nerve activity; SBRS, sympathetic baroreflex sensitivity; T2DM, type 2 diabetes mellitus.

**FIGURE 2:**
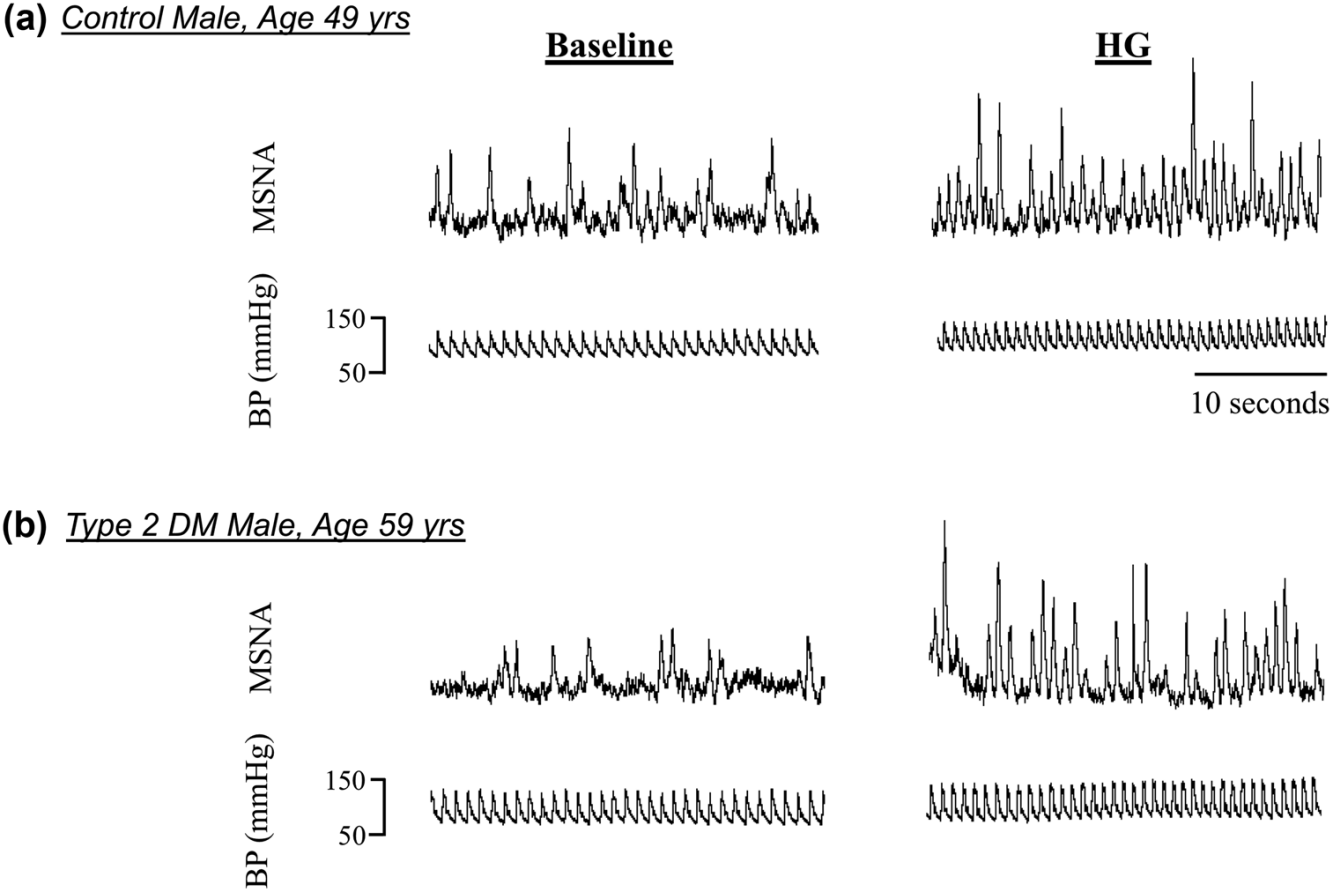
Representative recording of MSNA and BP in a control subject (2‐A) and a patient with T2DM. Abbreviations: BP, blood pressure; HG, handgrip; MSNA, muscle sympathetic nerve activity; T2DM, type 2 diabetes mellitus.

The SBP in T2DM was higher than in the control group (Table 1). Resting MSNA was recorded successfully in eight T2DM patients and 20 control subjects. Both MSNA‐BF and ‐BI at rest in the T2DM group were significantly lower than in the control group (Figure 1). Representative recordings of MSNA and BP in a control subject and a patient with T2DM are shown in Figure 2. Resting MSNA in the patient with T2DM was lower than that in the control subject; however, MSNA during HG was similar between the two subjects.

### 3.2 Resting HRV indices, CBRS and SBRS

Resting HRV was assessed successfully in 10 T2DM patients and in 18 control subjects (Table 1). The SDNN and LF (in milliseconds squared) in the T2DM group were significantly lower than in the control group (Table 1). Other HRV parameters were not different between groups (Table 1).

Resting CBRS was assessed successfully in 10 T2DM patients and in 16 control subjects, and resting SBRS was assessed successfully in eight T2DM patients and in 16 control subjects. The CBRS in the T2DM group was significantly lower than in the control group (Figure 1). The SBRS was not significantly different between groups (Figure 1).

By propensity score matching for BMI, nine subjects in the control group were matched to subjects in the T2DM group (Table 1). Even after propensity score matching, significant group differences in SBP, MSNA, LF and CBRS were observed (all *P* < 0.05; Table 1).

### 3.3 Relationships between glycaemic/insulin indices and resting MSNA, CBRS, SBRS and HRV

**FIGURE 3:**
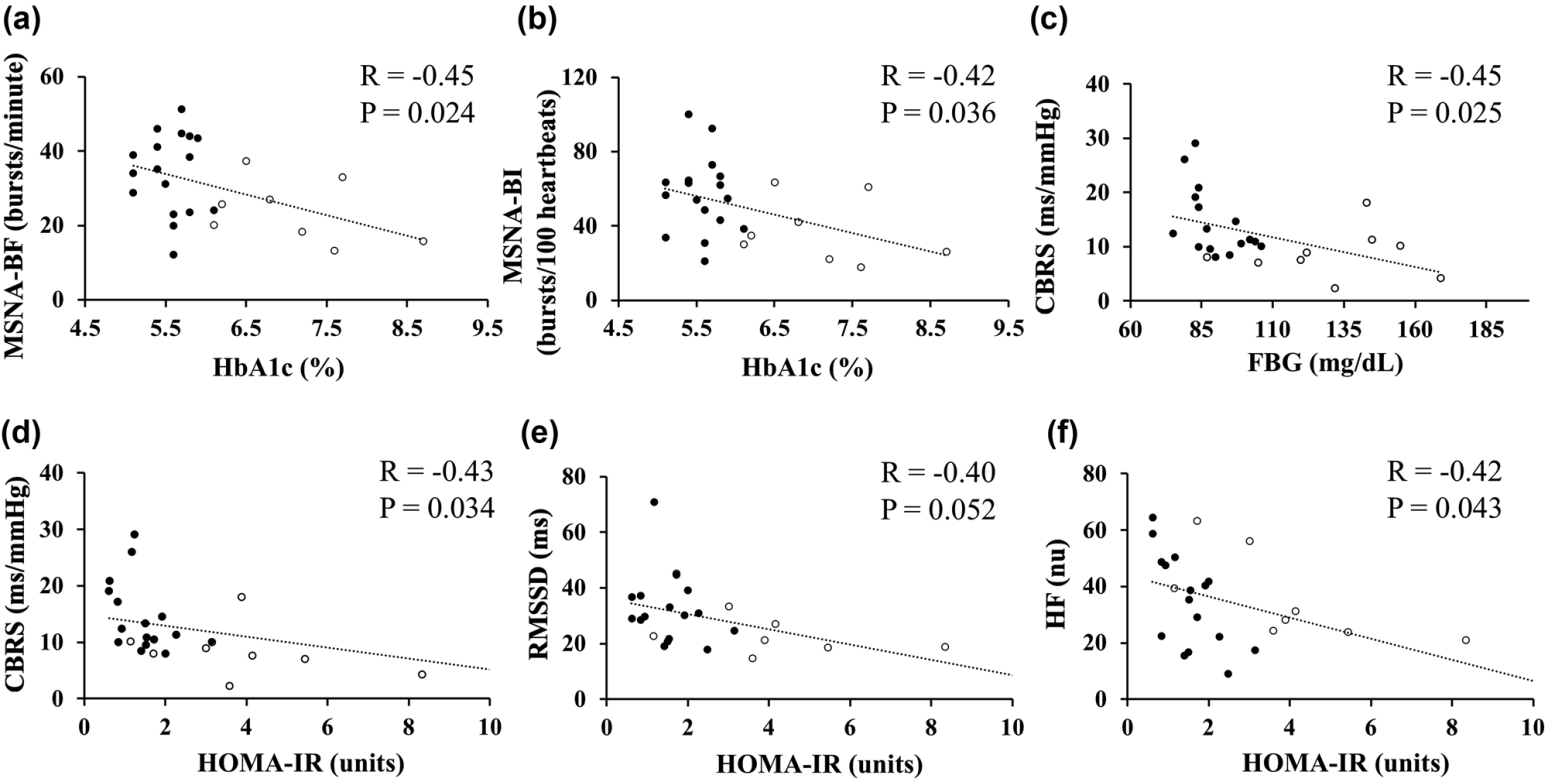
Relationships between glycaemic/insulin indices and MSNA‐BF (a), MSNA‐BI (b), CBRS (c, d), RMSSD (e) or HF (f) in all subjects. *R* indicates the correlation coefficient. Filled circles indicate control group individuals. Open circles indicate T2DM group individuals. Abbreviations: BF, burst frequency; BI, burst incidence; CBRS, cardiovagal‐baroreflex sensitivity; HF, high frequency; MSNA, muscle sympathetic nerve activity; RMSSD; root mean square of successive R–R interval differences.

With the data of all subjects, MSNA‐BF and ‐BI exhibited significant negative correlations with HbA_1c_ (both *P* < 0.05; Figure 3). Significant negative correlations were also observed between CBRS and FBG or HOMA‐IR (both *P* < 0.05; Figure 3). The SBRS‐BI and SBRS‐total MSNA were not correlated with the glycaemic/insulin indices (Table 3). Because the correlation between HOMA‐IR and insulin was very strong (*R* = 0.98, *P* < 0.0001), only HOMA‐IR was used as a parameter of insulin metabolism in regression analyses. However, the results are not provided; the results of the relationships between insulin and other parameters were the same as the relationship between HOMA‐IR and other parameters in this study.

**TABLE 3:** Relationships between glycaemic/insulin indices and parameters of autonomic function.

Parameter	All			Control			T2DM		
	FBG	HOMA‐IR	HbA_1c_	FBG	HOMA‐IR	HbA_1c_	FBG	HOMA‐IR	HbA_1c_
	*R*	*R*	*R*	*R*	*R*	*R*	*R*	*R*	*R*
MSNA‐BF, bursts/min	−0.37^a^	−0.41^b^	−0.45^*^	0.19	0.18	−0.04	−0.23	−0.77^l^	−0.39
MSNA‐BI, bursts/100 heart beats	−0.33	−0.39^c^	−0.42^*^	0.27	0.29	−0.08	−0.15	−0.75^m^	−0.22
CBRS, ms/mmHg	−0.45^*^	−0.43^*^	−0.25	−0.53^*^	−0.45^i^	0.06	0.09	−0.22	0.21
SBRS‐BI, bursts/100 heart beats/mmHg	0.15	0.32	0.05	−0.08	0.08	−0.41	0.44	0.80^n^	0.17
SBRS‐Total MSNA, units/beat/mmHg	0.23	0.26	0.13	−0.18	0.05	−0.47^j^	0.72	0.54	0.29
RMSSD, ms	−0.38^d^	−0.40^e^	−0.18	−0.25	−0.25	−0.16	−0.51	−0.51	−0.29
LF, ms^2^	−0.33	−0.18	−0.36^f^	−0.06	0.14	−0.20	−0.09	0.07	−0.05
HF, ms^2^	−0.34^g^	−0.31	−0.22	−0.40	−0.26	−0.23	−0.56	−0.48	−0.33
LF, normalized units	0.14	0.40^h^	−0.09	0.46^k^	0.61^*^	0.14	0.40	0.71^*^	0.10
HF, normalized units	−0.15	−0.42^*^	0.10	−0.51^*^	−0.66^*^	−0.18	−0.35	−0.68^o^	−0.06
LF/HF	0.002	0.25	−0.17	0.30	0.56^*^	−0.01	0.37	0.82^*^	0.03

Abbreviations: BF, burst frequency; BI, burst incidence; CBRS, cardiovagal‐baroreflex sensitivity; FBG, fasting blood glucose; HbA_1c_, glycated haemoglobin; HF, high frequency; HOMA‐IR, homeostatic model assessment of insulin resistance; HR, heart rate; LF, low frequency; MSNA, muscle sympathetic nerve activity; RMSSD, root mean square of successive R–R interval differences; SBRS, sympathetic baroreflex sensitivity; T2DM, type 2 diabetes mellitus.

* *P* < 0.05; ^a^ *P* = 0.075; ^b^ *P* = 0.054; ^c^ *P* = 0.069; ^d^ *P* = 0.06; ^e^ *P* = 0.052; ^f^ *P* = 0.070; ^g^ *P* = 0.097; ^h^ *P* = 0.056, ^i^ *P* = 0.080; ^j^ *P* = 0.093; ^k^ *P* = 0.074; ^l^ *P* = 0.073; ^m^ *P* = 0.086; ^n^ *P* = 0.060; ^o^ *P* = 0.064.

With the data of all subjects, RMSSD tended to be correlated with FBG (*P* = 0.060; Table 3) and HOMA‐IR (*P* = 0.052; Figure 3). The HF component (in normalized units) was negatively correlated with HOMA‐IR (*P* = 0.043; Figure 3).

For the data of the control group only, resting MSNA was not correlated with the glycaemic/insulin indices (Table 3). The SBRS was not correlated with the glycaemic/insulin indices (Table 3). The CBRS exhibited a significant correlation with FBG (*P* = 0.035; Table 3). The LF component (in normalized units) exhibited a significant correlation with HOMA‐IR (Table 3). The HF component exhibited a significant correlation with FBG and HOMA‐IR (both *P* < 0.05; Table 3). The LF/HF exhibited a significant correlation with HOMA‐IR (*P* = 0.024; Table 3).

For the data of the T2DM group only, MSNA tended to exhibit a negative correlation with HOMA‐IR (*P* = 0.073; Table 3). The LF/HF exhibited a significant correlation with HOMA‐IR (*P* = 0.012; Table 3). Other indices were not correlated with the glycaemic/insulin indices (Table 3).

### 3.4 Response to handgrip exercise in haemodynamic variables and MSNA

The MSNA response to HG exercise was recorded successfully in eight T2DM patients and in 19 control subjects. Handgrip exercise evoked significant increases in MAP (the effect of time, controls: *F* = 109.2, *P* < 0.001; T2DM: *F* = 17.1, *P* < 0.001) and HR (controls: *F* = 51.6, *P* < 0.001; T2DM: *F* = 28.7, *P* < 0.001) in both groups, and these increases were sustained during PEMI (Table 4). No group differences were observed in MAP and HR responses to HG exercise and PEMI (the effect of time and group interaction, *P* > 0.05; Table 4). Handgrip exercise and PEMI also evoked significant increases in MSNA‐BF (effect of time, controls: *F* = 26.9, *P* < 0.001; T2DM: *F* = 8.0, *P* = 0.005), MSNA‐BI (controls: *F* = 9.9, *P* < 0.001; T2DM: *F* = 4.3, *P* = 0.036) and total MSNA in both groups (Table 4). The MSNA responses to HG and PEMI did not differ between groups (effect of time and group interaction in both BF and BI, *P* > 0.05; Table 4).

**TABLE 4:** Blood pressure, heart rate and sympathetic nerve responses to handgrip exercise and postexercise muscle ischaemia.

Parameter		Baseline	Handgrip	PEMI	Two‐way MANOVA		
					Time	Group	Int.
MAP, mmHg	Control	89.4 ± 5.1	117.4 ± 7.7^*^	110.6 ± 9.2^*^ ^†^	*F* = 87.2	*F* = 1.9	*F* = 0.4
	T2DM	95.9 ± 8.3	120.9 ± 18.3^*^	113.8 ± 14.5^*^ ^†^	*P* < 0.001	*P* = 0.175	*P* = 0.686
	*P* (group)	0.013	0.466	0.470			
HR, beats/min	Control	60.5 ± 5.2	76.3 ± 10.4^*^	64.2 ± 8.5^*^ ^†^	*F* = 62.0	*F* = 1.4	*F* = 2.0
	T2DM	64.5 ± 9.9	76.9 ± 9.3^*^	69.8 ± 6.4^*^ ^†^	*P* < 0.001	*P* = 0.243	*P* = 0.148
	*P* (group)	0.149	0.870	0.073			
MSNA‐BF, bursts/min	Control	34.7 ± 9.7	48.4 ± 13.6^*^	42.9 ± 11.2^*^	*F* = 30.0	*F* = 4.7	*F* = 0.3
	T2DM	23.8 ± 8.5	40.2 ± 16.1^*^	34.0 ± 8.5^a^	*P* < 0.001	*P* = 0.041	*P* = 0.765
	*P* (group)	0.010	0.188	0.054			
MSNA‐BI, bursts/100 heart beats	Control	58.0 ± 18.6	67.0 ± 21.9^*^	67.5 ± 19.6^*^	*F* = 13.6	*F* = 5.3	*F* = 0.9
	T2DM	37.1 ± 17.1	52.8 ± 23.4^c^	49.1 ± 14.4	*P* < 0.001	*P* = 0.030	*P* = 0.432
	*P* (group)	0.011	0.143	0.025			
Total MSNA, units/min	Control	685.5 ± 250.9	1209.8 ± 532.9^*^	1039.9 ± 440.0^*^ ^†^	One‐way RMANOVA: Time		
	T2DM	484.8 ± 149.4	1111.1 ± 483.7^*^	862.3 ± 364.6^d^	*F* = 29.0, *P* < 0.001 (Control)		
	*P* (group)	NA	NA	NA	*F* = 9.3, *P* = 0.003 (T2DM)		

*Note*: Values are the mean ± SD.

Abbreviations: BF, burst frequency; BI, burst incidence; Group, the effect of group (T2DM or control); HR, heart rate; Int., interaction between time and group; MANOVA, mixed analysis of variance; MAP, mean arterial pressure; MSNA, muscle sympathetic nerve activity; NA, not applicable; PEMI, postexercise muscle ischaemia; RMANOVA, repeated measures analysis of variance; Time, the effect of time (baseline, handgrip and PEMI).

* *P* < 0.05 compared with baseline in the same group; ^†^ *P* < 0.05 compared with handgrip in the same group; *P* (group), *P*‐values between groups at the same time; ^a^ *P* = 0.053 compared with baseline; ^b^ *P* = 0.086 compared with baseline; ^c^ *P* = 0.089 compared with baseline; ^d^ *P* = 0.094 compared with baseline.

### 3.5 Relationships between glycaemic/insulin indices and the responses to HG exercise and PEMI

**FIGURE 4:**
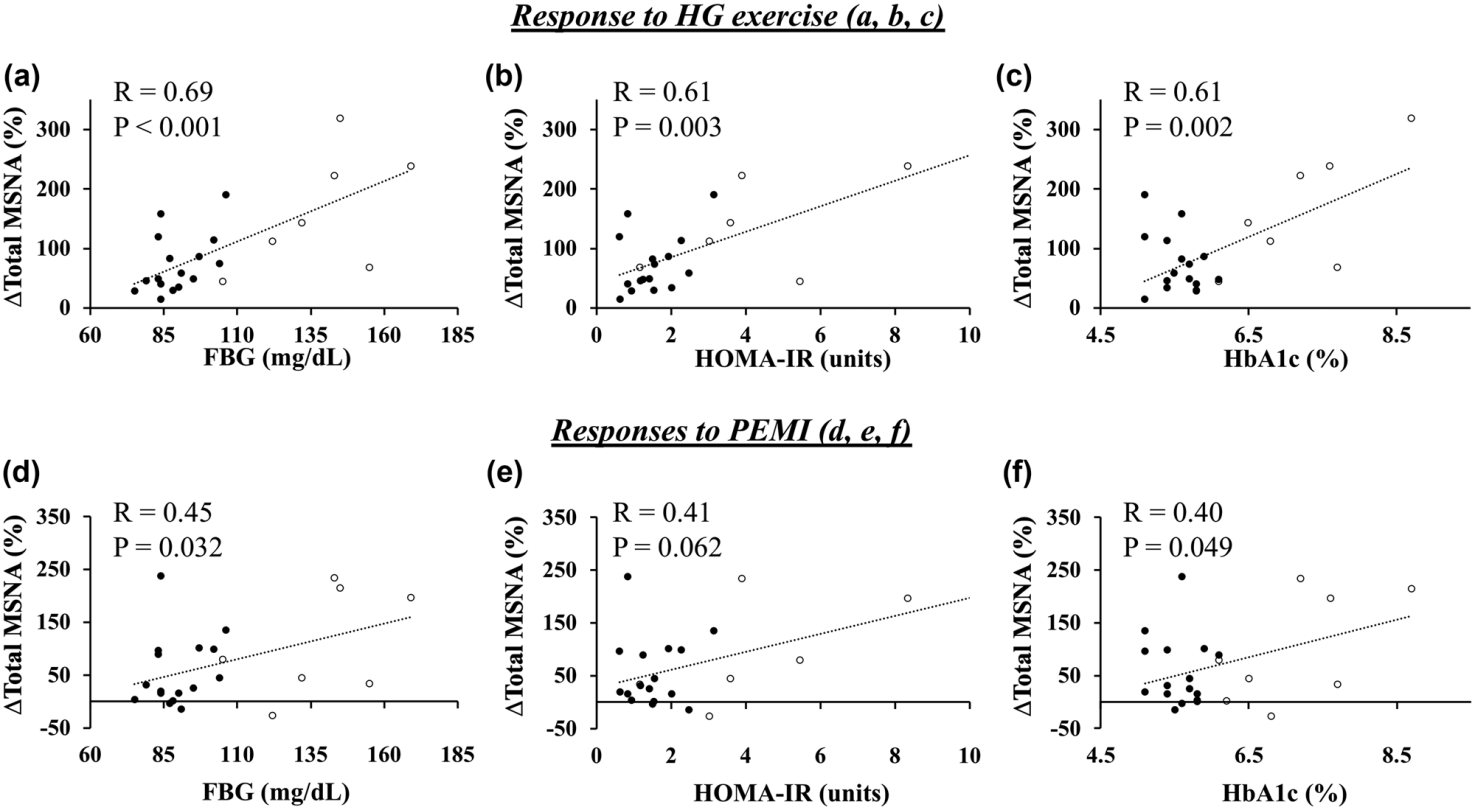
Relationships between ∆Total MSNA (as a percentage) by HG (a–c) or PEMI (d–f) and glycaemic/insulin indices in all subjects. Fille circles indicate control group individuals. Open circles indicate T2DM group individuals. Abbreviations: HG, handgrip; MSNA, muscle sympathetic nerve activity; PEMI, postexercise muscle ischaemia.

For the data of all subjects, ∆MSNA‐BI (in bursts per 100 heart beats) by HG exercise was significantly correlated with FBG (*P* = 0.022) and tended to be correlated with HbA_1c_ (*P* = 0.051). ∆Total MSNA (in units per minute) by HG exercise was significantly correlated with FBG (*P* = 0.039), and ∆Total MSNA (as a percentage) by HG exercise was significantly correlated with FBG (*P* < 0.001), HOMA‐IR (*P* = 0.003) and HbA_1c_ (*P* = 0.002) (Figure 4).

For the data of the control group only, ∆Total MSNA (in units per minute) was significantly correlated with FBG (*P* = 0.001) and HOMA‐IR (*P* = 0.012). ∆Total MSNA (as a percentage) tended to be correlated with FBG (*P* = 0.060). For the data of the T2DM group only, ∆Total MSNA tended to be correlated with HbA_1c_ (*P* = 0.072), and ∆Total MSNA was significantly correlated with HbA_1c_ (*P* = 0.021).

During PEMI, a significant relationship between ∆Total MSNA and FBG or HbA_1c_ was still observed for the data of all subjects (both *P* < 0.05; Figure 4). In the control group, ∆Total MSNA and ∆MAP (in millimetres of mercury) were significantly correlated with FBG (both *P* < 0.05). No significant relationships were observed between glycaemic/insulin indices and other variables during PEMI.

## 4 DISCUSSION

The main findings of the present study are as follows: (1) resting MSNA was significantly lower in T2DM patients than in control subjects; (2) across all study participants, resting MSNA was negatively correlated with HbA_1c_; (3) parasympathetic neural indices, expressed by HRV and CBRS, were negatively correlated with glycaemic/insulin indices across all subjects and for healthy control subjects alone, and differed from our hypothesis; and (4) the magnitude of the MSNA response to HG exercise in T2DM was preserved, and the MSNA response was positively correlated with the glycaemic/insulin indices across all subjects and even for control subjects alone.

### 4.1 Glucose metabolism and autonomic function at rest

A wide range of nerve fibres can be injured by the progression of T2DM (i.e., Aα, Aβ, Aδ and C fibres) (Vinik et al., 2013). The MSNA is measured directly from small and unmyelinated postganglionic C fibres (Vallbo et al., 2004). Thus, lower MSNA in our T2DM subjects could represent small‐fibre injury, which might become evident before large‐fibre injury that affects deep tendon reflexes and sensory/motor nerves (i.e., typical clinical manifestations of diabetic neuropathy) (Vinik et al., 2013). The SDNN and LF component can represent both sympathetic and parasympathetic indices of HRV (1996). In the present study, SDNN and LF in the T2DM group were also significantly decreased.

However, several previous reports showed increased MSNA in patients with T2DM (Huggett et al., 2005, 2003; Kobayashi et al., 2010; Straznicky et al., 2012). Hyperinsulinaemia has been considered one of the crucial mechanisms that can increase MSNA in T2DM by stimulating central sympathetic drive (Grassi et al., 2020; Huggett et al., 2003). In previous studies that showed increased MSNA in T2DM, untreated T2DM patients were included (Straznicky et al., 2012) or although treated T2DM patients were included, the high average values of insulin‐related indices (e.g., HOMA‐IR) in those studies might have affected the different MSNA results from our study (Huggett et al., 2005, 2003). As a matter of fact, in type 1 diabetes, which is less prone to hyperinsulinaemia, resting MSNA was normal or even decreased (Grassi et al., 2020). We speculate that in addition to hyperinsulinaemia, cardiovascular complications attributable to diabetes might play a prominent role in the chronic sympathetic activation in some patients with T2DM observed in those earlier studies. For instance, heart failure with reduced ejection fraction and coronary artery disease, common complications of T2DM, are known to increase resting sympathetic nerve activity (Floras, 2009; Gomes et al., 2010). We would like to note that patients with severe cardiovascular diseases, including coronary/cerebral artery disease, heart failure, pulmonary hypertension and renal failure, were excluded from the present study. We speculate that this could be one of the factors responsible for the lower resting MSNA observed in the present study. Nevertheless, we did include five T2DM patients with hypertension. It was reported that resting MSNA in T2DM patients complicated by essential hypertension was higher than in patients with T2DM without hypertension (Huggett et al., 2003). However, the BP in the patients with hypertension in the present study was well controlled (average SBP/DBP = 126/79 mmHg). Thus, the absence of significantly elevated BP in our T2DM subjects treated for hypertension might explain their MSNA data that were not elevated. Further studies are needed to clarify this issue.

The average resting MSNA was low in T2DM, while the average resting SBP in T2DM was higher than in the control group, although it was in the normal range. It is possible that non‐neural factors, such as structural vascular changes attributable to atherosclerosis, caused the difference in SBP between groups. However, a previous study showed increased sympathetic transduction without an increase in MSNA at rest in T2DM (Young et al., 2019). If sympathetic transduction is increased in T2DM patients in our study, it is possible that lower MSNA induced higher BP at rest in T2DM compared with control subjects. Future studies are warranted to assess sympathetic transduction in T2DM in more detail.

The parasympathetic neural indices in HRV (RMSSD and HF) in the T2DM group were not different from the control group, which differed from previous reports (Schroeder et al., 2005; Shah et al., 2019; Spallone, 2019). The control of T2DM was relatively good in our study (e.g., HbA_1c_, 6.8 ± 1.0%), which might explain the small group difference. In contrast, similar to previous studies (Schroeder et al., 2005; Shah et al., 2019; Spallone, 2019), significant linear relationships were observed between HF and HOMA‐IR in the data of all subjects, and between HF and FBG or HOMA‐IR in the control group. The negative correlation between parasympathetic indices and glycaemic/insulin indices still suggests that abnormal glucose/insulin metabolism could impair parasympathetic modulation of HR. Moreover, this relationship can be observed before clinical T2DM develops. Furthermore, LF/HF was positively correlated with HOMA‐IR in the control group. Thus, the decrease in parasympathetic modulation of HR is presumed to be significant compared with sympathetic activity, which would make for a sympathetic dominant condition in cardiac autonomic function in relationship to the severity of abnormal glucose/insulin metabolism. Interestingly, MSNA in the control group was not associated with any glycaemic/insulin indices. The vagus nerve, which mainly mediates parasympathetic activity, is the longest nerve in the autonomic nervous system (Ewing, 1996). Neuropathy caused by DM is reported to affect longer nerves at an earlier stage of the disease (Ewing, 1996). Thus, it has been speculated that diabetic autonomic dysfunction would exhibit a parasympathetic dominant impairment (Ewing, 1996). Our result of the significant relationship between glycaemic/insulin indices and HF or LF/HF but not MSNA in healthy control subjects might support this hypothesis.

Interestingly, only in the T2DM group data analysis, MSNA tended to be negatively correlated with HOMA‐IR. In contrast, in T2DM, LF/HF was positively correlated with HOMA‐IR. This result might indicate differences in cardiac autonomic dysfunction compared with the dysfunction of sympathetic nerves innervating peripheral vascular beds (i.e., a sympathetic dominant condition in cardiac autonomic function but decreased sympathetic nerve activity to peripheral vascular beds).

It is speculated that parasympathetic activity strongly affects CBRS (Higgins et al., 1973). In the present study, CBRS was lower in T2DM, which is consistent with several previous reports (Kück et al., 2020; Sakamoto et al., 2019). However, other reports (Holwerda et al., 2016b; Huggett et al., 2005) showed preserved CBRS in T2DM. In those reports (Holwerda et al., 2016b; Huggett et al., 2005), the Valsalva manoeuvre (Huggett et al., 2005) or vasodilator and pressor drugs (Holwerda et al., 2016b) were used to assess CBRS, and overweight control subjects (Holwerda et al., 2016b; Huggett et al., 2005) were included, which differed from the present study. It is known that CBRS is decreased in obese individuals (Alvarez et al., 2005). However, in our study, even matched by BMI, CBRS was decreased in T2DM, and linear relationships between CBRS and glycaemic/insulin indices were observed. Therefore, we postulate that the severity of abnormal glucose/insulin metabolism (e.g., HOMA‐IR) would affect CBRS regardless of whether diagnostic criteria for T2DM are met.

In contrast to CBRS, SBRS in the T2DM group was not different from the control group. The T2DM patients in our study were expected to be in a relatively early stage of T2DM (reported duration of the disease, 2.6 ± 2.3 years) and had no clinically obvious cardiovascular complications. The vagus nerve plays an important role in regulating HR (e.g., efferent signal to the heart) and CBRS (Higgins et al., 1973). Thus, it seems plausible that CBRS decreased in relationship to glycaemic/insulin indices from a very early stage of abnormal glucose/insulin metabolism, similar to the HRV parasympathetic indices. Baroreflex control of postganglionic MSNA is mediated by a pathway that is different from the vagus nerve and might be less affected. Thus, we speculate that the effects of T2DM on SBRS might occur later in the progress of T2DM than the effects on CBRS. Interestingly, unlike in the control subjects, in the T2DM group, SBRS‐BI tended to be correlated with HOMA‐IR (*P* = 0.06) in our study, which might indicate decreased SBRS in severe T2DM. Further studies, including T2DM patients with a wide range of disease severity, are warranted.

### 4.2 Glucose metabolism and the sympathetic nerve activity response to exercise

Although resting MSNA was lower, the magnitude of the MSNA response (∆MSNA) to HG exercise and PEMI in the T2DM group was preserved. Importantly, across all subjects, the MSNA response to HG was correlated with glycaemic/insulin indices. Moreover, this relationship was also observed in healthy control subjects. The results suggest that the MSNA response to HG is increased with progressively abnormal glucose metabolism. The average value of the MSNA response to exercise in the T2DM group was not different from that of healthy subjects in our study, which might be because diabetes was well controlled in this group. If the glucose/insulin metabolism was worse, it would be expected that the MSNA response to exercise would exceed that in non‐DM individuals. These results are consistent with previous reports that showed an elevated sympathetic response to exercise in T2DM (Holwerda et al., 2016a; Vranish et al., 2020) and an elevated pressor response in prediabetic individuals (Hotta et al., 2020). However, as far as we know, no study has evaluated the relationship between glycaemic/insulin indices and the MSNA response to exercise in healthy individuals without diabetes. Also, our comprehensive assessment of resting MSNA and the MSNA response to exercise showed a significantly different relationship between glucose/insulin metabolism and resting MSNA or the MSNA response to exercise. We speculate that this is because of the differences in nerve fibres that relate to the response. In addition to efferent C fibres, the response to exercise reflects the afferent Aδ, C (i.e., group III and IV) nerve activities innervating skeletal muscles (Grotle et al., 2020; Kim et al., 2019; Teixeira & Vianna, 2022). It is suggested that insulin potentiates the response to mechanical or chemical stimuli in small dorsal root ganglion neurons and in group III and IV muscle afferents (Hori et al., 2022; Hotta et al., 2019; Mizuno et al., 2021). Furthermore, abnormal activation of group III and IV afferent nerves induced by abnormal metabolism in exercising muscle (e.g., oxidative stress) in T2DM was reported to be associated with an exaggerated pressor reflex (Grotle et al., 2020; Ishizawa et al., 2021). Interestingly, even in type 1 DM, which showed lower resting sympathetic nerve activity, exaggerated sympathetic responses to exercise were observed (Grotle et al., 2019, 2020; Ishizawa et al., 2020). However, further studies are needed. As shown in the present study, it should be recognized that the relationship between glucose metabolism and resting MSNA differs from the relationship between glucose metabolism and the MSNA response to exercise in T2DM.

### 4.3 Limitations

There were several limitations in this study. First, T2DM patients in this study had mild/controlled diabetes of limited duration. Therefore, we do not know whether our results (e.g., lower resting MSNA) apply to severe T2DM patients with longer disease duration, even if their serum insulin level and cardiovascular complications are well controlled. Second, we could not show which parameter is crucial among glycaemic/insulin indices for the relationship with autonomic function. Because the number of subjects was relatively small, it was difficult to perform multivariate analyses in this study. Third, detailed evaluations of neuropathy, such as nerve conduction velocity measurements, were not performed because we focused on the patients before the manifestation of symptomatic neuropathy. Therefore, some asymptomatic patients with neuropathy might have been included. Fourth, we could not exclude the effects of medication. For example, many patients used statins, which might have affected MSNA (McGowan et al., 2013). However, another report suggested that MSNA was unchanged by statin use in patients with T2DM (Young et al., 2019). In our study, MSNA was not different between T2DM patients who were using statins (*n* = 6) or not (*n* = 5) (e.g., BF with statins vs. without statins, 24.8 ± 5.8 vs. 22.0 ± 13.3 bursts/min; *P* = 0.768). In this study, T2DM patients with hypertension who were already receiving medication were included. Therefore, we cannot exclude the effect of hypertension and medications on the results. However, it should be noted that in these patients, hypertension was well controlled. Moreover, resting MSNA in T2DM patients with controlled hypertension was not different from that of T2DM patients without hypertension (T2DM without hypertension vs. T2DM with controlled hypertension, 24.8 ± 12.1 vs. 22.8 ± 4.2 bursts/min; *P* = 0.774). Nevertheless, we acknowledge these limitations. Finally, this was a cross‐sectional study. We could not confirm whether parasympathetic and sympathetic nerve activity decreased during the progression of T2DM. Future studies are warranted to examine this issue.

### 4.4 Perspective

In our study, parasympathetic dysfunction and an augmented sympathetic response to exercise were associated with abnormal glucose/insulin metabolism even before the development of T2DM. These results are consistent with the notion that prediabetes is associated with cardiovascular disease risks (Færch et al., 2014) and suggest the importance of early therapeutic interventions for abnormal glucose/insulin metabolism. Also, based on our data, we speculate that parasympathetic autonomic dysfunction precedes sympathetic autonomic dysfunction at rest, which can cause a sympathetic dominant condition in cardiac autonomic function. Indeed, a favourable effect of vagal stimulation in a rat model of T2DM was recently reported (Payne et al., 2022). To prevent future cardiovascular events, neuromodulatory intervention on parasympathetic nerves in addition to conventional therapy for diabetes deserves to be explored.

## 5 CONCLUSION

The present results demonstrated that MSNA and parasympathetic modulation of HR at rest were decreased in patients with T2DM. Abnormal glucose/insulin metabolism was associated with changes in resting MSNA, parasympathetic modulation of HR, and cardiac baroreflex control in patients with T2DM and healthy individuals. However, these changes were not parallel, and baroreflex control of MSNA in these patients was preserved. The results also showed that glucose/insulin metabolism contributed to the MSNA response to exercise even before the development of overt diabetes. These observations might suggest the importance of a comprehensive assessment of autonomic function from different pathways (e.g., heart and resistance vessels) to understand the severity of autonomic dysfunction accurately.

## AUTHOR CONTRIBUTIONS

Lawrence I. Sinoway and Jian Cui conceived and designed the research. Urs A. Leuenberger, Rachel C. Drew, Cheryl Blaha, Jonathan Carter Luck and Jian Cui performed the experiment. Takuto Hamaoka, Matthew Murray, Cheryl Blaha, Jonathan Carter Luck and Jian Cui analysed data. Takuto Hamaoka, Urs A. Leuenberger, Matthew Murray, Jonathan C. Luck, Lawrence I. Sinoway and Jian Cui interpreted the results of experiments. Takuto Hamaoka prepared figures. Takuto Hamaoka, Urs A. Leuenberger, Lawrence I. Sinoway and Jian Cui drafted the manuscript. Takuto Hamaoka, Urs A. Leuenberger, Lawrence I. Sinoway and Jian Cui edited and revised the manuscript. Takuto Hamaoka, Urs A. Leuenberger, Rachel C. Drew, Matthew Murray, Cheryl Blaha, Jonathan Carter Luck, Lawrence I. Sinoway and Jian Cui approved the final version of the manuscript and agree to be accountable for all aspects of the work in ensuring that questions related to the accuracy or integrity of any part of the work are appropriately investigated and resolved. All persons designated as authors qualify for authorship, and all those who qualify for authorship are listed.

## CONFLICT OF INTEREST

The authors declare no conflicts of interest.

## Data Availability

The datasets generated and analysed during the present study are available from the corresponding author upon reasonable request.
